# Development of a Secure Website to Facilitate Information Sharing in Families at High Risk of Bowel Cancer—The Familyweb Study

**DOI:** 10.3390/cancers13102404

**Published:** 2021-05-16

**Authors:** Selina Goodman, Heather Skirton, Leigh Jackson, Ray B. Jones

**Affiliations:** 1 College of Medicine & Health, University of Exeter Medical School, RILD Building, Barrack Road, Exeter EX2 5DW, UK; L.Jackson2@exeter.ac.uk; 2 School of Nursing & Midwifery, University of Plymouth, Drake Circus, Plymouth PL4 8AA, UK; heatherskirton@outlook.com (H.S.); ray.jones@plymouth.ac.uk (R.B.J.)

**Keywords:** bowel cancer, genetic testing, familial cancer, relatives, website, information sharing, genetic diagnosis, communication, cancer surveillance, Lynch syndrome

## Abstract

**Simple Summary:**

Families with an inherited high risk of bowel cancer may struggle to share information about their diagnosis. This means that relatives are not always aware of their increased risk of cancer or able to access screening for the early detection of cancer. Through this study, we aimed to help such families by creating a website where patients could share confidential information with their relatives securely online. Following a survey and telephone interviews with affected individuals, the content of the website was developed to suit the needs of families. Website function was tested with patients to check feasibility and acceptability. Most participants wanted more information to support their adaptation to the diagnosis and help inform their relatives. This study demonstrates how health professionals can improve access to genetic testing and cancer screening in families at high risk of cancer, thus reducing morbidity and mortality.

**Abstract:**

Individuals with pathogenic variants in genes predisposing to bowel cancer are encouraged to share this information within their families. Close relatives at 50% risk can have access to bowel cancer surveillance. However, many relatives remain unaware of their vulnerability or have insufficient information. We investigated the feasibility and acceptability of using a secure website to support information sharing within families at high risk of bowel cancer. Patients (*n* = 286) answered an anonymous cross-sectional survey, with 14 participating in telephone interviews. They reported that the diagnosis had a profound effect on them and their family relationships, and consequently desired more support from health professionals. Website content was created in response to the preferences of survey and interview participants. Reactions to the website from 12 volunteers were captured through remote usability testing to guide further refinement of the website. Participants welcomed the opportunity to store and share personal information via the website and wanted more information and help informing their relatives about the diagnosis. Important website topics were: healthy lifestyle; genetic testing; and how to talk to children about the diagnosis. A website providing online access to confidential documents was both feasible and acceptable and could translate into increased uptake of cancer surveillance, resulting in lower morbidity and mortality in these families.

## 1. Introduction

An inherited vulnerability to cancer affects many people worldwide [1,2]. Typically, syndromes that confer a high risk of specific tumours are inherited in an autosomal dominant manner, where the first degree relatives of someone with a pathogenic variant are at 50% risk of having the same genetic variant [1]. Identifying those at risk and enabling them to access appropriate diagnostic screening (such as colonoscopy) is an effective strategy to reduce morbidity and mortality [3,4]. Consequently, it is important to investigate and then implement interventions that can facilitate cancer predisposition cascade testing and cancer surveillance [5]. Relatives at risk of cancer first need to be aware of the familial diagnosis, then understand the implications of that for themselves and have the ability to access testing and surveillance. Access may be facilitated by documentary evidence of risk; therefore, we sought to make an intervention that could address those factors: awareness, understanding and evidence of risk.

Screening for colorectal cancer in high risk individuals is an example of personalised preventative medicine. Whilst screening by colonoscopy can benefit the individual by reducing their risk of invasive cancer [3,6], personal choice is not possible until someone is informed of the options available to them. It has been demonstrated that the uptake of genetic testing and subsequent cancer screening is influenced by many factors, both organisational and personal. Disease registries can help organise screening for patients that are known to them [5,7,8] and specialist clinics can provide coordinated advice and screening, but clinical guidelines are not always applied in practice [9,10]. Evidence suggests that genetic testing in relatives at risk of bowel cancer is suboptimal [5,11,12]. This causes real concern on several levels: those at risk may not be receiving appropriate surveillance and treatment; conversely, those relatives who have not inherited the vulnerability may be having unnecessary colonoscopy, and calculations of cost-effectiveness for testing in bowel and endometrial cancers (which are guiding health policy in the UK and Australia) would be undermined [13,14]. The relatively low uptake of genetic testing may in part be due to family members not wishing to have genetic testing [15], but contributing factors could be the limited information provided for relatives in clinical practice [16], the cost of testing [17], the absence of coordinated care and uncertainty in affected relatives [18].

Several groups have sought to develop interventions to improve communication in families to improve awareness and appreciation of cancer risk [19]. These studies have taken place across different health systems (in the UK, Europe, Australia and the USA) [20,21,22,23,24,25,26,27,28,29,30,31], but common to all of them is the premise that patients could benefit from guidance about how to approach their relatives and explain to them about the diagnosis. In addition, it has been suggested that patients benefit from receiving information that is written for them or tailored to their specific needs [27,32,33]. In the USA, a website (https://kintalk.org/) (accessed on 27 February 2021) was created in order to encourage and support people with Lynch syndrome or HBOC to share information about the diagnosis with their relatives. This website has the strapline “*empowering families through communication and education”* and provides a secure document sharing function that we wished to emulate. Although the Kintalk website has been cited on several occasions [34,35,36,37], we could find no published evidence of its efficacy or acceptability with users at the time of our study. We were also mindful that the experiences of patients in the USA could be substantially different to that of patients in other countries due to the differences in the delivery of healthcare [38]. For example, the cost implications of having a genetic test will differ between the UK and USA and might be an inhibiting factor in the USA [36,39]. Conversely, although testing is free through the National Health Service (NHS), patients are not routinely given copies of their test results in the UK [16]. Consequently, we were interested to find out whether access to digital copies of documents provided by their health professional would be important to patients.

Informing relatives of a familial diagnosis can still be a challenging experience even with appropriate information and guidance on how to disseminate information [40,41]. A personal conflict can be felt between someone’s rational decision to pass on information to family members and their emotional unease at the potential disruption to family relationships that might ensue [42,43]. We believe that it is unusual for patients to overtly refuse to pass on information to their close relatives, but it is more common that disclosure is incomplete [44,45]. Whatever the context, we would argue that what is important is to sufficiently empower individuals to act in a way that is consistent with their beliefs and ideals [46]. With the increasing use of technology in communication [47], it would appear logical to offer documents in both hard copy and digital format as this might be particularly helpful to younger, less mobile or geographically distant relatives [48].

Defining what is deemed to be an effective intervention may not be confined to the simple use of numbers of relatives contacted. Other measures have showed that sharing information about a diagnosis was associated with the patients’ perception of how the news would be received and whether the informant felt this was within their control [25] consistent with the Theory of Planned Behaviour (TPB) [49]. Under the TPB, “perceived personal control” is only one determinant of intention, which is also modified by people’s “attitudes” and “subjective norms”; these taken together lead to people’s intentions to a given behaviour. The controllability of certain behaviours is specific to individuals because of what they perceive as the difficulty of performing the behaviour will depend on both external factors (e.g., opportunity to exercise) and internal factors (e.g., knowledge of what foods are beneficial). We suggest that this theory can also be applied to issues such as how relatives might seek referral to genetics services and cancer surveillance.

An alternative theoretical framework which can be applied in this context is the Health Belief Model (HBM) [50], where cancer risk reduction might be considered a strong “perceived benefit” in response to the “perceived threat” to health posed by an inherited vulnerability to cancer. Improved understanding of someone’s vulnerability though better access to relevant literature or professional support could provide a “cue to action”, although this could be dependent on the person’s self-efficacy, as found in relation to dietary change in a study of patients with Lynch syndrome [51].

However, it can be difficult to quantify the effectiveness of risk communication interventions because counting the number of people who choose not to access a service is very challenging. Outcomes vary but have included counting the proportion of relatives referred to genetic services [22] and patients’ own assessments of the number of relatives they have informed [25]. Both these approaches are subject to error as they are reliant on the patient understanding who needs to be informed, while relatives may wait an indefinite time to seek testing or choose not to do so [12]. Consequently, we chose to investigate, as a proof of concept, a method of disseminating digital documents to patients that was designed to help them pass on information to their family members.

## 2. Materials and Methods

### 2.1. Design

We used a pragmatic mixed methods approach [52] which enabled us to gain a more thorough understanding of the issues relating to the delivery of patient-specific information in a healthcare setting [53,54]. With the intention of gathering information with both breadth and depth, we used a convergent mixed method design [55]. This utilised a questionnaire alongside qualitative interviews with a purposive sample of participants drawn from survey respondents [55,56].

We first carried out a systematic literature review [57] in order to find the existing evidence on methods of electronic communication used by health professionals to support families communicating about a health issue. This literature review was carried out across 10 electronic databases for the period of January 1990 to December 2017. Although 3587 articles were retrieved and 105 assessed in full text format, only one article by Bowen and colleagues [58] was identified that met the criteria, thus demonstrating the dearth of evidence in this area. However, these authors did emphasise the importance of alerting family members to a shared health threat (risk of melanoma), which would enable relatives to make informed choices about accessing screening or further advice [58,59].

### 2.2. Aims and Objectives

The aim of this research was to investigate whether a secure website could support families at an increased risk of bowel cancer to share information with their relatives. This research was performed as part of a doctoral study at the University of Plymouth, UK [60]. In order to achieve this, our four main objectives were:

1. Explore the perspectives of patients, including their experiences of how they received information about the familial diagnosis themselves;
2. Explore patients’ views about a secure website that provided a platform for patients to share documents about their diagnosis with their relatives;
3. Investigate the feasibility and acceptability of sharing electronic documents regarding a familial diagnosis securely online using the purpose built website;
4. Test the website’s function and acceptability with research participants [60] (p. 98).

### 2.3. Ethical Approval

Ethical approval was given by the Health Research Authority (HRA) South West Research Ethics Committee (IRAS reference 15/SW/0250) and by the University of Plymouth Faculty of Health Research Ethics Committee (reference 15/16-477).

### 2.4. Phase 1: Anonymous Cross-Sectional Survey

A cross sectional survey was used [60], administered both in paper format and online (using SurveyMonkey https://www.surveymonkey.com/) (accessed on 27 February 2021) [61]. The questionnaire was designed to elicit the views of a broad cohort of individuals living with a genetic vulnerability to bowel cancer. The text of the questionnaire is presented in Appendix A. In this phase, we wanted to explore participants’ experiences of being informed of the diagnosis and how that information was shared in the family. Data security of this sensitive information was ensured through (SG’s) password protected access to the responses submitted via SurveyMonkey, a platform considered sufficiently secure for health related surveys [62,63,64] and approved by the Ethics Committee within the study protocol [60].

#### 2.4.1. Inclusion and Exclusion Criteria for Phases 1 and 2

Participants were over 17 years old and were either part of a family considered to have an increased risk of bowel cancer due to their family history, where a genetic variant had been found which conferred a high risk of bowel cancer, or because they had been diagnosed with a cancer which was due to an inherited vulnerability, such as Lynch syndrome [65]. The key aspect of eligibility was whether they or their relative had been advised to have regular bowel screening by colonoscopy. Participants were not eligible if they had only received their diagnosis (genetic or cancer) within the last three months or if they were not competent in reading and speaking English. Language competence was assessed by local recruiting clinicians, or through self-selection as Participant Information Sheets (PIS) were provided in English only.

#### 2.4.2. Recruitment to Phases 1 and 2

Recruitment to the survey was through (1) NHS clinics at six recruitment sites with a paper copy questionnaire and (2) online via advertisements and links at four charity websites (Lynch Syndrome UK, Bowel Cancer West, Beating Bowel Cancer and FAP Gene Support Group). Information leaflets, the PIS and invitation letters were distributed in the Endoscopy Clinic at an NHS Hospital Trust. In addition, invitation letters and appropriately headed PIS were distributed to eligible patients at the five NHS clinical genetics services in England and Wales.

#### 2.4.3. Data Collection Phase 1 Survey

Data were collected through completion of the survey questionnaire, either online or on paper. Participants completing and returning the anonymous survey questionnaire were interpreted as giving assent to taking part, so explicit consent was not required for this aspect of our study. Nominal categorical data were collected using fourteen multiple choice questions. There were also six open questions with free text boxes inviting more detailed responses or elaboration to some answers. In addition, one question had a Likert type scale [66] giving a range of options between “very unhelpful” and “very helpful” to different formats for receiving information.

#### 2.4.4. Data Analysis Phase 1 Survey

Qualitative data from free text responses were coded and analysed for recurrent themes [67,68] by the first two authors, using NVivo qualitative data analysis software [69]. The quantitative data from the cross-sectional survey [70] were analysed using descriptive statistics in Microsoft Excel (2016 version Microsoft, Redmond, WA, USA) and using SPSS software (IBM SPSS statistics version 22 IBM Corporation, Armonk, NY, USA). Pearson’s Chi Square or Fisher’s exact tests were used to give a measure of association between categorical variables. The responses to the Likert type questions were analysed as ordinal data using descriptive statistics and Chi-squared as a measure of association [66].

#### 2.4.5. Recruitment Phase 2 Telephone Interviews

This phase of the study used a nested sampling design [71] where there was an invitation at the end of the survey questionnaire for participants to take part in further research. Tear off slips were laid out so that they could be separated from the questionnaire and returned using the Freepost address. This meant that participants in Phase 2 could have known about their diagnosis, or the diagnosis in the family, for some time and were therefore drawn from the same population of patients as for Phase 1.

#### 2.4.6. Sampling and Data Collection Telephone Interviews

Semi-structured telephone interviews were used to collect data from a purposive sample of respondents to the survey, with maximum variation for age and educational qualification and with equal numbers of men and women [56]. Participants had received a PIS and consent forms prior to interview and their verbal consent was also taken prior to interview. The digital recordings of the interviews were then transcribed to allow coding and subsequent analysis by content and theme [67]. Participant confidentiality was maintained through the use of pseudonyms and redacting any potentially identifiable place names or data.

#### 2.4.7. Data Analysis Phase 2

Data analysis was based on a qualitative thematic analysis approach using both deductive and inductive coding [67,72,73]. This was performed in order to develop information most suited to the needs of the potential recipients and define concepts of interest or concern. Coding was primarily deductive through content analysis and descriptive coding, focusing on how the participant had been informed of their risk, how health issues were communicated in the family and which topics they would like more information about. Secondary analysis was inductive, focusing on the development of theories about what helped or hindered communication in the family over health issues. Coding of transcripts was carried out by two coders (S.G. and H.S.) independently and any differences in coding were resolved through discussions until a consensus was reached.

### 2.5. Website Development

**Figure 1:**
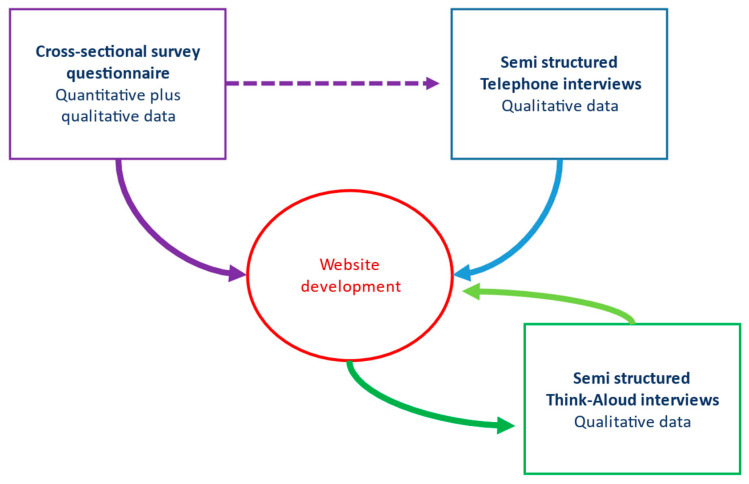
Interaction between the different phases of the study and website development.

**Figure 2:**
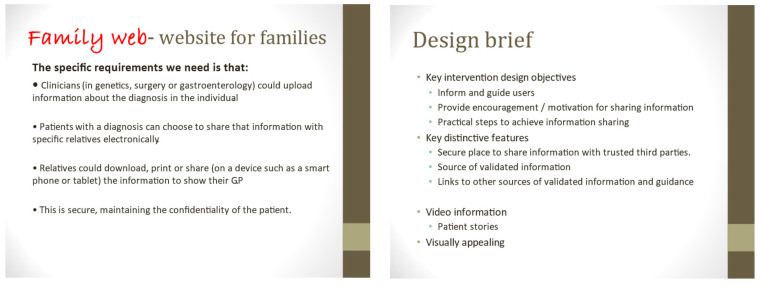
Two slides from the brief given to the web developer as a presentation.

The Family Web website (https://www.familyweb.org.uk/) (accessed on 27 February 2021) was developed in response to the findings from Phases 1 and 2 of the study (Figure 1), which mainly guided the content of the information resources. The structure of the website was created by a web development company (https://www.modernwebsites.co.uk) (accessed on 27 February 2021). The brief had been to create a website that would function as an alternative means of sharing health information securely with patients and their at-risk relatives (Figure 2). It was therefore designed as a secure database where health professionals, or patients, could upload documents in a range of digital file formats (e.g., pdfs, jpeg, word files) and set up secure online links to these documents which could then be shared with relatives via email.

The level of security was achieved through a very secure database with multiple levels of encryption, which was well within what was required by NHS Information Governance [74,75] to ensure the website’s acceptability to clinical users in the UK. In addition, all connections to the website used the Hypertext Transfer Protocol Secure (https) protocol with a verified level of security with Secure Sockets Layer (SLL) certificates, and all communication between the website and the browser was encrypted.

**Figure 3:**
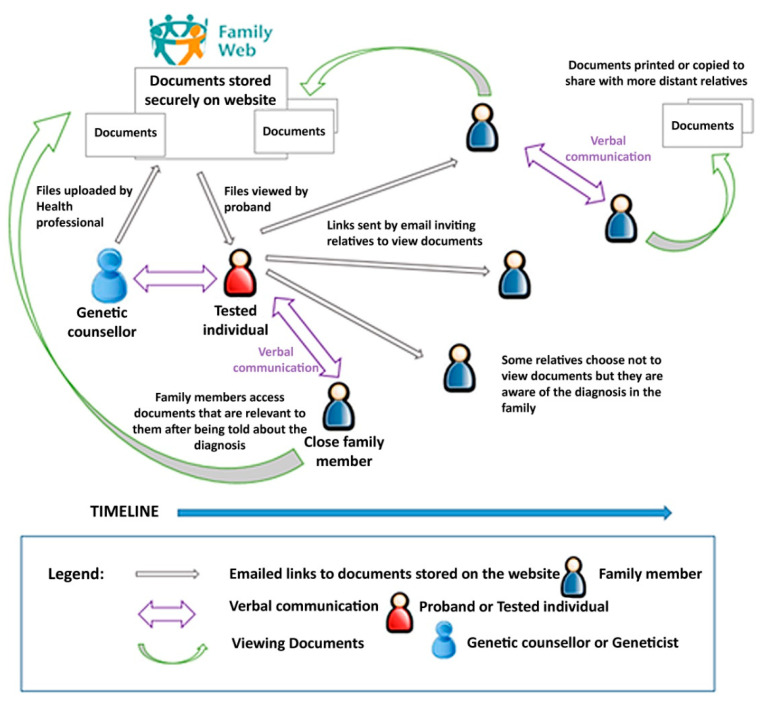
Document sharing function of the website [60].

The aim of this study was to test the usability and acceptability of the website. Data were collected regarding website activity in terms of users viewing the open access information pages. In addition, an activity log captured anonymous information about those users who created “member” accounts and how many times they shared documents with other people. This in turn allowed the activity log to collect data on which members’ documents were viewed by their relatives (Figure 3).

### 2.6. Phase 3: Think-Aloud Interviews to Test Website Function

#### 2.6.1. Data Collection

**Figure 4:**
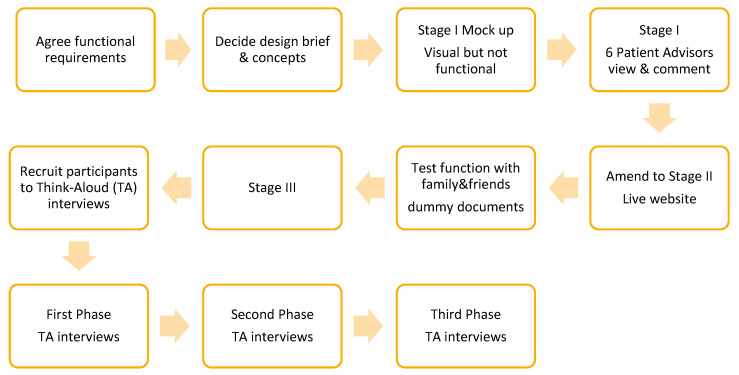
Iterative phases of the website development.

The website was developed (Figure 4) and tested through an iterative series of twelve Think-Aloud interviews [76] with eligible participants. These were semi-structured interviews conducted through the online video conferencing platform GoToMeeting [77]. During each Think-Aloud interview, participants navigated through the website and voiced their thoughts, at times prompted by the interviewer (SG). Each interview was recorded to enable analysis and reflective notes were made during the interview, and immediately afterwards, noting any issues or technical difficulties that had occurred. This type of dynamic interview was used to capture each participant’s interaction with the website alongside their verbalisation [78].

The criteria for eligibility were almost the same as quoted above (Section 2.4), but in contrast to Phases 1 and 2 the people who were eligible for Phase 3 were more recently diagnosed. We wished to recruit people who had only learnt about their genetic vulnerability to cancer within the last 24 months. This was because we wanted to interview people who might still be in the process of sharing information with their relatives. Recruitment was through the six existing NHS recruitment sites (only name, contact number and email address were provided) and respondents to the online survey who had offered to be interviewed via the reply slip. Following receipt of a PIS and consent form, potential interviewees were contacted by telephone to explain the process of a Think-Aloud interview and consent; signed forms were returned prior to interview.

Twelve Think-Aloud interviews were conducted, initially with four participants who had no prior experience of the website. Subsequently, eight further interviews were recorded after other participants had been given the opportunity to first explore the website in their own time.

#### 2.6.2. Phase 3 Data Analysis

Video recordings were replayed several times, enabling SG to view them while making notes about each participant’s comments and reactions to different elements of the website. A matrix was created linking participant comments with their context and allowed comparison between participants for each section of the website. Illustrative quotes were identified through verbatim transcription of phrases. This was an iterative process which identified themes and enabled the website to be refined in response to earlier participants’ comments and actions.

## 3. Results

### 3.1. Phase 1 Survey

Of 286 people who participated in the survey, 217 were women (77%). There was a broad age distribution of participants with 68 (24%) under 40 years old and 145 (51%) were 50 years or older. Most responses were received from the online survey: 183/286 (64%).

Over half, 165/284 (58%), reported that they had never been diagnosed with cancer, but 87 had experienced bowel cancer and 32 another type of cancer. The majority of participants (249/286, 87%) reported that a genetic test was available in their family.

The largest proportion of participants (43%) had first been informed of the familial diagnosis by a relative, most often their mother, while 28% were told by a genetics specialist and 20% by another specialist. In addition, 88 (31%) were the index case in their families with the main responsibility for passing on information to their other family members. The survey question did not ask about specific numbers of relatives who had been informed of the diagnosis but instead asked if “all”, “most”, “some” or “none” were aware of the risk of cancer. Of those 88 index case participants, only three reported that none of their relatives were aware, but only 29 (33%) indicated that “all” of their relatives knew about their increased risk.

When comparing responses and distinguishing between those people who had been educated to above or below degree level, there was an association between lower educational attainment and receiving no supporting information at the time of their diagnosis (Fisher’s exact 10.24; *p* < 0.5). When asked about what, if any, information had been received, 140 (49%) indicated “general information”. However, 40/124 (32%) of participants had not received any written information when they had first learnt about the diagnosis through their family member.

Despite this, most participants indicated that they had felt supported at the time of their genetic diagnosis or when they first learnt about the increased vulnerability to cancer in their family. Satisfaction with support was most often reported (104/148) if they had received information from a health professional. Nonetheless, the majority, 193/256 (75%), also wanted more information and support, ideally provided through a follow-up appointment or via email. One comment in the free text responses summarised this well:

*“Informal advice about how to broach this subject with relatives, some of whom I do not see often and who are not local to my area. The ‘To whom it may concern’ letter is rather too formal in my opinion”*.

Survey Participant 49 [60]

When given options of which topics (“talking to children”, “healthy lifestyle”, “helping relatives who live abroad”, “genetic testing” and “other”) participants wanted more information about, most participants 140/286 (49%) indicated that they wanted more advice on how to have a “healthy lifestyle”, followed by “genetic testing” (44%) and “talking to children” (34%).

### 3.2. Phase 2 Telephone Interviews

**Figure 5:**
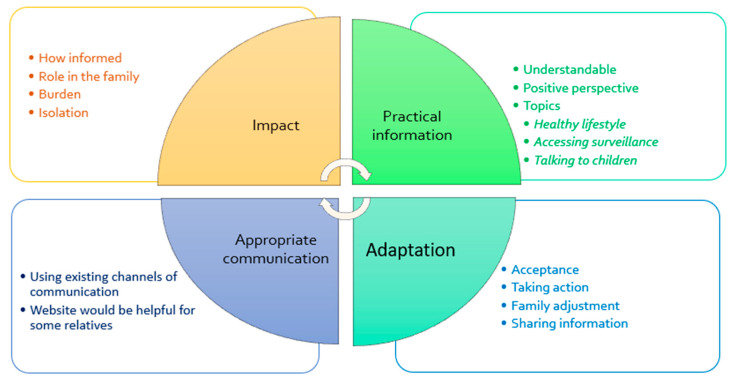
Suggested interactions between major themes [60].

Data were collected through telephone interviews with a purposive sample of volunteers (six men and eight women) in order to understand some of the difficulties encountered using the website and preferences for information of people of different ages and both sexes. This number of interviews was sufficient to reach saturation of themes [79]. The volunteers had ages ranging from 20 years to 68 years and 12 out of 14 of them had a diagnosis of Lynch syndrome. Four major themes of “impact of the diagnosis”, “adaptation to the diagnosis”, “appropriate communication” and “practical information” were identified and Figure 5 below illustrates how these themes might interact.

For the purpose of this paper, we will focus on the themes of “practical information” and “appropriate communication” as most relevant to the technological aspects of facilitating communication.

Participants wanted more information to build on their understanding of the familial diagnosis and how best to mitigate the effects in practical ways. Advice on how a healthy diet and lifestyle could reduce their risk of cancer was commonly mentioned and included the use of aspirin [80,81]. Other topics which were frequently raised were: how to spot the early signs of cancer, how to talk to children about the diagnosis, the risks of different cancers associated with their genetic variant and how to access appropriate cancer screening.

The participants who had already shared information about the diagnosis with their relatives described using a variety of different methods of communication. The type of communication depended on what was feasible, likely to suit the recipient and was considered appropriate for the task. One participant had taken on the role of family co-ordinator and explained that he had used Facebook messenger when contacting his niece, while another printed out a letter to send to her elderly parent. In most cases, participants described how they had initially broken the news about the genetic diagnosis during a visit, so in person, or by telephone. This personal approach was considered ideal, but some recognised that physical or emotional distance might influence what they did.

Many interview participants reported seeking information from the Internet and they considered the opportunity to store and share their own information via a secure website as being useful, as long as that was appropriate for the recipient. One person (Interview Subject 8) said:

*“I would use whatever was appropriate to the person I was talking to. So, you know, if I was trying to explain to my father then I would use a patient information leaflet. If I was trying to explain to my nieces, then I would say “Look at this website””*.

[60]

The content of written information was described as important by some, with the clinic summary letter often described as too personal, detailed and clinical to be shared. However, for several people the post clinic letter appeared to be the only written information they had received. This aligned with other responses where participants explained that written information needed to be accurate but simple. This requirement impacted both on the patients themselves and on their relatives, as some participants described how they felt they had insufficient understanding to be confident explaining the condition to others.

Talking more specifically about how they could use a secure website for sharing information, one woman envisaged how her brother might find that accessing the information online might suit him while he was having trouble coming to terms with the familial diagnosis:

*“once he gets to that point where he feels “I can face it now” he can go and have a look and he can find the information but he needs to do it in his own time”*.

(Interview Subject 12) [60]

### 3.3. Results Phase 3 Think-Aloud Interviews

The Think-Aloud interviews captured comments (all names are pseudonyms) in relation to initial impressions of the website in the first iteration with four users (Oliver, Luke, Jane and Freya); experiences of the website after having access two days prior to interview with four other users (Theo, Mike, Annie and Harry); and reflections particularly around the document sharing function with access a week before the interview with a further four users (Mark, Stella, Jenny and Keith). The participants in these interviews were a purposive sample of interview volunteers with seven men (average age 52 years) and five women (average age 45 years). The youngest participant was Freya (25 years) and the oldest was Harry (69 years). The participants took part in a location of their choice, visiting the website while being interviewed via an online video conferencing platform (GoToMeeting) [77].

**Figure 6:**
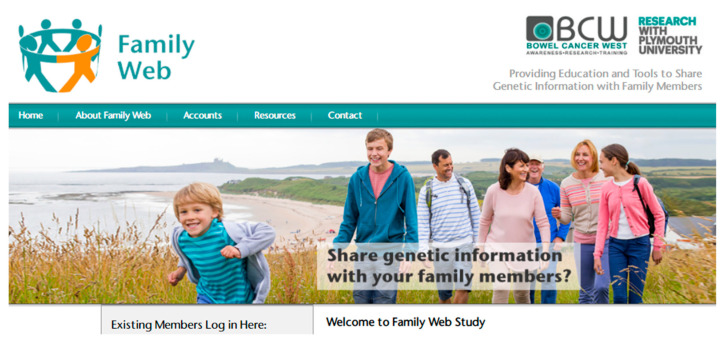
Screenshot of the Family Web homepage banner.

Initial impressions of the website were positive—the first four interview participants all said how much they liked the look of the website (Figure 6); they were interested in how it worked but they did not immediately understand the function of the website:

*“I think the content looks really good and the way it is laid out is really good, it is really clear*…

*… It’s not instantly clear what it is regarding… is this just about people with bowel cancer awareness?”*.

Luke (#2)

All these participants were drawn to the open access resources and two were enthusiastic about how these were laid out in sections under the title “Your Journey”. They particularly appreciated the information provided about the importance of a healthy diet in the “Living your Life” section:

*“This is my favourite page of it all because these are the questions that I have had to go away and find answers to myself”*.

Oliver (#1)

One element that caused confusion to Freya (#4) was how users to the website were invited to create free accounts but this was divided into “patient”, “family member” or “professional” account types. Freya explained that she was not sure she was a “patient” as she had not had cancer, and she also related to being a “family member”.

**Figure 7:**
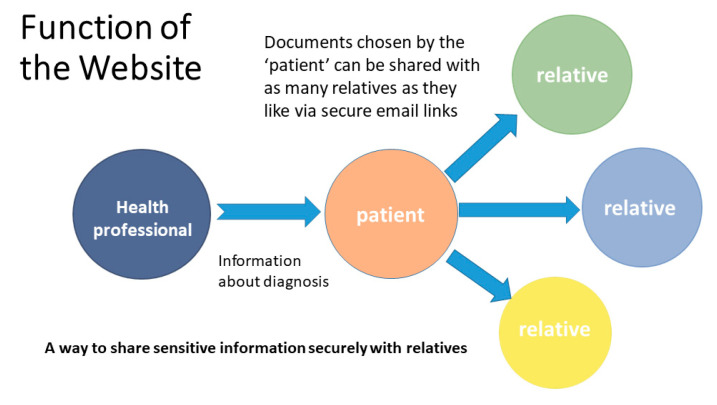
Graphic on “About Family Web” page to represent document sharing function [60].

In response to comments by two of the participants, the graphic above (Figure 7) was added to the website. The text on the homepage was also simplified with bullet points introduced in order to provide a list at a glance and links with quick access to different parts of the website.

Another area that received criticism was the section on bowel screening, which was considered by some to have insufficient detail. One participant explained how screening was the most important aspect of her diagnosis:

*“How to get the right screening is quite important. Obviously diet, lifestyle, alcohol but the screening is what is most important. People want to know what they have to do.”*

Jenny (#11)

Along with information about other ways to reduce the risk of cancer, one participant said how he wanted others to be aware that colonoscopy was *“nothing to be afraid of”* Mike (#6). Following these comments, a short video about colonoscopy was added to the site. Other material that was changed following the interviews were: addition of information about aspirin reducing risk of cancer; removal of a diagram of biological mechanisms linking certain foods to cancer; provision of more information about the sign-up process to make it clearer.

Table 1 below provides a matrix of the specific changes made to different parts of the website in response to reactions given in the Think-Aloud interviews, with interviews numbered in chronological order denoted by #symbol and number.

How the inherited condition was presented was seen as critical by some participants, who explained that in order to feel motivated to share the news of the diagnosis they needed to feel that there was some benefit for their relatives. Freya (#4) said that the positive messages needed more prominence and drew attention to the potential protective effect of diet and exercise, saying:

*“I would put that right at the top. Something like.. healthy diet, exercise, everything that is good for everyone but you are at higher risk and it makes even more of a difference for you.”*

Freya (#4) also specifically explained that she needed to have hope and know the benefits of her genetic status in order to have the motivation to act on that information, rather than just experience it as bad news. This in turn would enable her to share those explanations of benefit to other people in her family.

**Figure 8:**
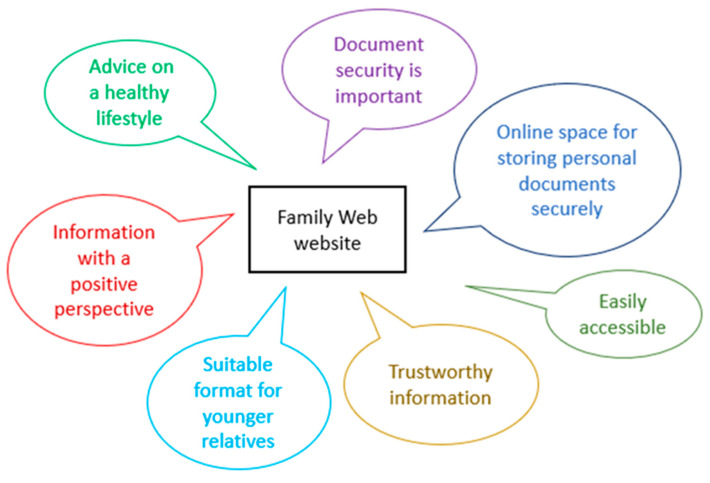
Illustrating some of the elements of the website that users liked.

Two users in the third phase of Think-Aloud interviews were particularly enthusiastic about the opportunity the website gave them to give their family members access to documents at a time of their choosing. One user described how her teenage grandchildren might feel uncomfortable talking to her about their bowels: she also thought that they might want to read the information when they could be on their own. Some of the key elements that the users described as important are illustrated above (Figure 8). Overall, the Think-Aloud interviews demonstrated that the website provided functions that were welcomed by users, its appearance was pleasing and there were only minor adjustments needed.

## 4. Discussion

This study provides new evidence that patients would use a web-based file sharing facility, such as the FamilyWeb website. It demonstrates the proof of concept that a website that is used by patients to share digital documents with their family can be feasible. We have demonstrated the content and functionality of such a website that we were able to design and which we were able to adapt in response to patient users. We believe that this research has made a significant contribution to understanding what factors influence the dissemination of information in families in this context. Participants sought information which was tailored to their needs, which was offered through uploading patient-specific documents. This finding is consistent with other studies [82] which have found that patients expected a range of information sources and at a level of complexity that suited their particular needs. Whether use of this type of technology could translate into increased cascade testing and screening would depend on whether it was utilised by health care providers [83], patients and their relatives.

### 4.1. Practical Advice and the Theory of Planned Behaviour

We suggest that users might experience greater “perceived behavioural control” over their likelihood of developing cancer when they have access to information that enables them to act towards reducing their risk, such as dietary advice or aspirin use. In this context, the Theory of Planned Behaviour [34,83,84] can be used to describe determinants of behaviour and could predict why receiving practical advice was reported to be important by many of the participants. Improved understanding and feelings of hope attributed to greater “perceived behavioural control” could motivate patients to share documents with their relatives. In addition, our results indicated that some participants anticipated that sharing information in this way would be approved of by their relatives, such as the website user who believed her teenage grandchildren would prefer to view documents online.

Conversely, some of the participants in this study reported feeling uncertain about what they should tell their family members or they lacked confidence in their own understanding of the relevant issues, so “lacked perceived behavioural control”. The information they had received was often not considered appropriate for the task so they welcomed the opportunity to have further appointments with their genetics health professional or receive more information (via an email or a website). It was also clear from all phases of the study that participants valued practical advice and were seeking ways to reduce the uncertainty they felt about the impact of their diagnosis on their lives. What they wanted was practical information written in a simple and accessible format that had content which was directly relevant to them.

### 4.2. Information That Is Trustworthy and Accurate

Evidence suggests that patients want tailored information from trustworthy sources [84,85]. Participants to this study wanted more support from health professionals to access appropriate cancer surveillance and they looked for a positive message about the genetic diagnosis to share with their relatives. We suggest that when information comes from a trusted source, it provides users with greater confidence. If someone has an inaccurate or insufficient understanding of the implications of the familial diagnosis for their own health [86], it is less likely that they will access the appropriate screening or share that information with other relatives. Providing open access resources via a website alongside information which demonstrates its authenticity (e.g., NHS badging) provides the opportunity to create a trustworthy source of guidance which can be updated as new evidence emerges. This means that a website of this type with ongoing curation could be used as a powerful tool in education and dissemination of advice to people living with an increased risk of cancer. All of the open access resources could be considered as “educational materials” as they were written with the intention of informing readers on aspects relating to the familial diagnosis, such as how to “share the news” of the diagnosis, what might reduce their risk of cancer through adopting a healthy lifestyle and information about bowel cancer and colonoscopy that might be useful to relatives in a “before diagnosis” section. Previous research in families with Lynch syndrome in the USA had shown associations between receiving educational material and seeking genetic advice. The retrospective questionnaire-based study would have been liable to errors of recall but showed that significantly more relatives who received educational materials went on to seek advice from a health professional compared to those that received no materials [87].

### 4.3. Positive Framing of Information

Many participants appeared to feel motivated to help their relatives access appropriate healthcare and several groups have already described how patients wish to protect their family members [29,43,88]. Users to the website said how they liked the positive stance of the resources. Information that gave hope and indicated a benefit to knowing genetic status was perceived as a potentially motivating factor by some participants. This finding concurs with McAllister’s theory of engagement, within which she suggested that someone’s negative response to knowing their high risk of cancer was ameliorated if they understood the benefits of bowel screening [89]. Our finding could also link to theories proposed by Lafreniere and colleagues [90], who found evidence in families with HBOC that the initial reactions of family members influenced how someone might attempt to communicate with other relatives; if they were listened to and received positively this reinforced the action and encouraged them to further communication within the family.

However positive the message, the process of attempting to inform other family members about the familial diagnosis can be greeted with anger, denial or disagreement [43,91]. We acknowledge that not all relatives may wish to know about the genetic vulnerability to cancer in their family [15]. Choosing not to seek genetic counselling or have genetic testing can be a legitimate choice and a valid protective mechanism for some relatives. The Health Belief Model [50,92] describes how an individual’s behaviour can be conceptualised in terms of the perceived threat that the condition presents to them and whether they experience any conflict between their self-efficacy and the perceived barriers or benefits of behaviour change. Since someone’s assessment of the level of threat is likely to be dependent on their understanding and their self-efficacy, we would argue that it remains imperative that relatives have access to clear, factual information which is written to explain the benefits of knowing their genetic status as well as the medical implications. The methods for providing that information need to be in whatever format is most appropriate for that person, so ideally available in several formats (leaflet, letter, digital document, video, social media). One motive for this research was the increasing rates of bowel cancer diagnosed in younger people [93], and we would argue that it is therefore particularly important to use online methods of disseminating information in a manner likely to promote empowerment.

### 4.4. Study Limitations

Creation of The FamilyWeb website was an attempt to address some of the problems associated with family communication, using a secure site. This created a facility for health professionals to upload reports or letters to provide an encrypted link for patients to access their own reports and letters. In turn, patients could choose to share specific documents with as many relatives as they wished. Clinicians at the recruitment sites did not actively engage with this process of uploading documents because they had delegated recruitment to their research teams, so a next step in this research would be to test this function within clinical practice with health care professionals providing patients with copies of reports via the website. Developing a robust protocol to investigate the use of the website with health professionals might be informed by the Technology Acceptance Model, where key variables of “perceived usefulness” and “perceived ease of use” should be assessed [94]. We also recognise that the transmission of information as a proxy indicator of communication is too simplistic; sharing information may occur as a single event (transmitter–receiver) but this does not take into account the perspectives of the receiver [95]. Another element of technology adoption is likely to be trust in the security of the site. While the security of our website was adequate for this study, we recognise that maintaining security is an ongoing process and that trust would develop over time. Further work would be needed in collaboration with NHS IT departments to ensure security was acceptable and develop trust.

Another limitation was that participants were asked for their postcode as a proxy for geographical location within the UK but ethnicity was not captured as part of the demographic data. The greatest proportion of survey participants were recruited through a Genetics Service which serves an ethnically diverse population, but we have no evidence of how accessible or appropriate the website was to different ethnic groups, so this needs to be addressed in future research.

### 4.5. Research Recommendations

We did not seek to test the efficacy of the website in disseminating documents; however, this could be achieved through further website development. The research reported here demonstrated a proof of principle that a website of this kind could function as a means of providing patients with access to their confidential documents (clinic letters, test results, screening reports) if they were uploaded to the site by their clinician.

The current build of the website would allow the collection of data on the number of documents shared with relatives and how many of those relatives accessed each document, in addition to the number of views of each page or resource. The website also logs the times that users and relatives access the website, distinguishing between different relatives by assigning a unique number to each person (since their identity is unknown). This means that it would be straightforward to calculate the proportion of relatives who actively engage with the website and view the documents for which they have been sent links. However, it would not be possible to determine the relationship and level of risk of each family member contacted without requesting such data through a user survey.

Currently the website does not recognise if a new user who creates an account was previously someone who was sent an encrypted link by their family member, but this function could be built in to enable the website to track the cascade effect of using the website to disseminate information within a family. Therefore, this website, or a Personal Health Record system which incorporated the same functions, could be used as a tool to collect such data within a larger study.

In Finland, Denmark, Australia and the Netherlands, initiatives have already been taken to investigate the feasibility and acceptability of contacting at-risk relatives directly rather than relying on a family-mediated approach to disseminate information about genetic diagnoses [8,12,96,97]. Although direct contact was not envisioned as a use for the website, health professionals could send encrypted links directly to a patient’s relatives via email with the prior consent of that patient. This approach has already been suggested in the context of familial hypercholesterolaemia as a way of reducing the burden on patients by using software to inform relatives directly [98].

We focused on facilitating communication in families at increased risk of bowel cancer, but it would be a logical step to investigate whether the same approach could be used across other genetic conditions. We would echo the sentiments of Schwiter et al. in their recent mini-review of communication and cascade testing uptake [19] that web-based content could be made available to families in a format that could be engaging, but also affordable and sustainable. Their finding supports that of other authors [99,100,101] that tailored information is more likely to be seen as relevant and is particularly beneficial to patients. Consequently, we would intend to collect data on how tailored information can be optimised in a future pilot study of the website within a clinical context.

The content of the Family Web website could be changed or extended to be relevant to many different patient groups, but we do also acknowledge that the functionality of this type of secure database is already built in to some existing Personal Health Record systems. What is lacking is robust research evidence to demonstrate their efficacy at disseminating information in families because this would not have been regarded as a primary function at their inception. However, in the wake of the COVID-19 pandemic, there may be an increase in research which utilises technology to assist in communication due to the rapid adoption of novel methods of service delivery during the crisis [102].

## 5. Conclusions

This research has provided a proof of principle that providing patient-specific documents securely online is both feasible and acceptable to patients. In the past, the growing use of digital communication (email, video call, social media) has not been reflected in health communication due to concerns about security and confidentiality. In publicly funded health systems, such as the NHS, patients are reliant on the health professionals who order their tests and typically they do not receive copies of their reports; consequently, individuals may not possess the material that could empower them [103]. We are more optimistic that there has been an accelerated adoption of technology in health since the COVID-19 pandemic [104], which could translate into increased use of innovations such as our website to facilitate communication in families.

## Figures and Tables

**Table 1:** Changes made to the website linked to suggestions given in TA interviews [60].

Interview	Area of Site	Suggestions for Improvement	Changed
#4, #6, #9, #10	Home page	Need to say what people can do, what they can find on the site, at a glance list showing relevance to users.	Bullet points added with short cuts to provide quick access to different parts of the site.
#4, #7 #2, #4	About Family Web	Banner picture too big, you have to scroll down to see text. More explanation needed about what website function is.	Some pictures removed or reduced in size. Graphic created to show function of the website.
#4	Account information	Banner picture unnecessary, just obscures information	Banner picture removed
#7, #12 #1, #6	Patient sign up	Problems with creating username Need more directions for sign-up	Added statement that username must be in lower case letters Text added to explain account activation needed before use. More detail added to the instructions.
#9 #4, #5	Document sharing	No partner as option in drop down list of relatives Blue folder icon meaningless	‘Partner’ added as an option to drop-down list Change blue folder icon to ‘Share Files’
#4, #6, #6, #10, #11 #2 #7	Living your life	Disliked biological mechanisms graphic. Screening very important so wanted more information about it. Information about aspirin & CAPP3 Some pictures distract from the text.	Biological mechanisms graphic moved to another page. Added more information about colonoscopy Information about aspirin added Picture reduced in size
#6	Useful websites	More links to different sites wanted	More websites added, including link to CAPP3 study and NICE guidelines re tumour testing.

## Data Availability

The datasets generated for this study are available on request to the corresponding author.

## Appendix A

1. Who first told you that there was a risk of bowel cancer in your family? Please tick one: □ Your doctor (General Practitioner “GP”) □ Specialist doctor (e.g., surgeon, gastroenterologist, oncologist, etc.) □ Genetics specialist (e.g., medical doctor or genetic counsellor) □ Another healthcare professional □ Your relative, can you tell us who? (e.g., mother, brother, cousin?) Other person, please tell us who? (e.g., friend or charity advisor?) □ Can’t remember Please answer the next questions (2, 3 & 4) only if you were told about the increased risk by a healthcare professional 2. Did you feel well supported at that time? Yes/No/Not sure 3. Please give suggestions below if you think that this could have been done better? 4. Are you the first person in your family to be told that there is an increased risk of cancer in the family? Yes/No/Don’t know 5. Please can you tell us what written information you received when you were told about your risk of cancer? Tick all that apply □ None received □ General information about the condition □ Specific information about your family □ A copy of your family tree indicating who had cancer □ A copy of your family tree showing who could have bowel screening □ A ‘Dear Relative’ or ‘To Whom it May Concern’ letter to give to your relatives □ Other—Please give details 6. Did you get the information you wanted? □ None of what I wanted □ Some of what I wanted □ Most of what I wanted □ All of what I wanted 6a. If you didn’t get all the information you wanted at that time, what other information might have been helpful? Please tell us… 7. If you have found additional information about the shared risk of cancer in your family, who provided that information? Tick all that apply □ Your doctor, surgeon or other health professional □ Other relatives □ Friends □ Support group or charity meeting □ Internet website □ Social media □ Library 8. If you found out more information via the Internet, what websites or social media were particularly helpful? Please give details below: 9. Would you like to receive information in other ways? Yes/No/Don’t know If yes, would this be □ Via a website □ By Email □ Social media □ In a follow-up appointment □ Other—Please state We would like to know if other forms of information for patients could make it easier to share information in the family. Below, we ask you to think about other ways that your doctor or genetic counsellor could give you information. Then please can you rate how helpful these might be to you and your relatives? 10. Please indicate how helpful you think this would be for the different ways of getting information by making a cross on each of the scales below: a. A paper leaflet which has general information about an increased risk of bowel cancer, the implications for relatives and the screening available? b. A secure email which has more specific information about your increased risk, the implications for your relatives and the screening advised? c. A password protected website which has more specific information about your increased risk, the implications for your relatives and the screening advised ? d. A follow-up appointment in the hospital clinic where you are given specific information about your increased risk, the implications for your relatives and the screening advised? e. A follow-up telephone call where you are given specific information about your increased risk, the implications for your relatives and the screening advised? 11. What issues would you like more information about? Please tick all that apply □ Talking to children □ Healthy lifestyle □ How can I help my relatives who live abroad □ How to find out more about genetic testing □ Other issues Please give us more details about any of the issues that concern you in the box below: 12. How many of your relatives are aware of the increased risk of cancer in the family? So, as far as you know □ All □ Most (please tick the one which applies) □ Some □ None □ I don’t know 13. If you have experienced any difficulties sharing information with your relatives about the increased risk can you tell us what those difficulties were? Please give details below, or pass on to the next question if there were no difficulties 14. Can you suggest ways in which your health professional could help you or your family more? Please give suggestions below.
Final page of the questionnaire was of demographic questions
